# Relative Age Effects in Mathematics and Reading: Investigating the Generalizability across Students, Time and Classes

**DOI:** 10.3389/fpsyg.2016.00679

**Published:** 2016-05-17

**Authors:** Katharina Thoren, Elisa Heinig, Martin Brunner

**Affiliations:** ^1^Division of Evaluation and Quality Management in Education, Department of Education and Psychology, Free University BerlinBerlin, Germany; ^2^Division of Early Childhood Education, Department of Education and Psychology, Free University BerlinBerlin, Germany; ^3^Berlin Brandenburg Institute for School Quality ImprovementBerlin, Germany

**Keywords:** relative age effects, generalizability, immigrant students, multilevel modeling, large-scale assessment

## Abstract

A child's age in comparison to the age of her or his classmates (relative age) has been found to be an influential factor on academic achievement, particularly but not exclusively at the beginning of formal schooling. However, few studies have focused on the generalizability of relative age effects. To close this gap, the present study analyzes the generalizability across students with and without immigrant backgrounds, across three student cohorts that entered school under a changing law of school enrollment, and across classes. To this end, we capitalized on representative large-scale data sets from three student cohorts attending public schools in Berlin, the capital of Germany. We analyzed the data using a multilevel framework. Our results for the overall student sample indicate relative age effects for reading and mathematics in favor of the relatively older students in Grade 2 that become somewhat smaller in size in Grade 3. By Grade 8, relative age effects had vanished in reading and had even reversed in favor of the relatively young in mathematics. Furthermore, relative age effects were not found to be systematically different among students with and without immigrant backgrounds, student cohorts, or across classes. Taken together, these results empirically underscore the broad generalizability of the findings as found for the overall student population and replicate the pattern of findings on relative effects as identified by the majority of previous studies.

## Introduction

Effects of age on various outcomes such as academic achievement (e.g., Fertig and Kluve, 2005; Puhani and Weber, 2005), educational attainment (e.g., Angrist and Krueger, 1992; Black et al., 2011), or earned wages (e.g., Mayer and Knutson, 1999; Bedard and Dhuey, 2006) have been addressed in many studies in education and economic research. One widely-recognized way of investigating this question is to study how the relative position of a child with regard to her or his age has an effect on achievement when the average age of a class or learning group is higher or lower (Gold et al., 2012). A major advantage in focusing on these so-called relative age effects is that here, one makes use of the naturally occurring variation of birth months among students who are enrolled in school at the same time. In most OECD countries, education administrations define one cut-off date for school enrollment, stipulating that children who have reached a defined age (usually 6 or 7 years) by that date are eligible for school. This procedure usually results in an age range of up to 12 months within a school entrance cohort that coincides with differences in maturity as well as different learning experiences prior to school enrollment in preschool or in the family. In fact, studies have shown that relatively older students might show between 0.45 and 0.49 of a standard deviation better results in literacy at the beginning of formal schooling (Crone and Whitehurst, 1999; Gold et al., 2012). These findings illustrate the large heterogeneity among students in one class, particularly in the early elementary school grades. Accordingly, the importance of studying the relationship of relative age on educational outcomes also results from the fact that it affects children and teachers in their everyday lives.

The overarching goal of the present study is to address the generalizability of relative age effects on students' achievement. To this end, we study relative age effects (a) across two important academic domains (reading and mathematics), (b) in different subgroups of students by contrasting general effects with effects among students with and without immigrant backgrounds, (c) across time by investigating relative age effects for different school entrance cohorts (2004, 2005, and 2011), and (d) across classes by analyzing whether our results are applicable to different learning groups or classes. To this end, we capitalize on a unique set of population and large-scale data from students in the capital of Germany: Berlin.

## Literature review

### Relative age effects

Most of the studies analyzing achievement related relative age effects at the beginning of formal schooling have found that relatively older students perform better academically than relatively younger students: For German students, Gold et al. (2012) find effects in favor of older students for reading in Grade 1 but similar achievement already by Grade 2. A study by Kawaguchi (2011) states effects in favor of older students in Grade 4 mathematics in Japan. However, a study by Crone and Whitehurst (1999) for the United States finds relative age effects in favor of the relatively older in literacy skills at the beginning of formal schooling, but no relative age effects for reading skills by the time students reach the end of first grade. Analyzing relative age effects in secondary school, most studies have found effects favoring the older: Cobley et al. (2009) report positive effects for the relatively older for mathematics but not for English in Grades 7, 8, and 9 in England. Strøm (2004) finds these effects for reading among 15-year-olds in Norway, Fredriksson and Öckert (2006) for mathematics, Swedish and other domains in Grade 9 in Sweden, and Kawaguchi (2011) for mathematics in Grade 8 in Japan. There is, however, also evidence from the United States that relative age effects might vanish or even reverse in favor of the relatively younger in the course of students' school careers (Lincove and Painter, 2006—using a composite score of reading and mathematics). In summary, findings with relative age effects in favor of relatively older students dominate at the beginning of formal schooling. Over the course of primary and secondary education, evidence for the persistence of relative age effects seems to be mixed. Nonetheless, most studies that focus on long-term outcomes state at least decreasing effects (Stipek, 2002).

What mechanisms may explain differences in achievement between relatively younger and older children? Differences in cognitive development between younger and older children already occur before the beginning of formal schooling (Musch and Grondin, 2001) because older children (a) are simply older and on average more mature (Stipek, 2002; Bedard and Dhuey, 2008), and (b) will have had more time to receive institutionalized support while attending preschool facilities (Gold et al., 2012). After school entry these differences can persist or even increase because (c) the curriculum typically targets the average developmental state of students in one grade, which might be to the disadvantage for the youngest of the class (Elder and Lubotsky, 2009). Moreover (d) teachers' behavior in the classroom might differ for older compared to younger children. For instance, they might have higher expectations of older children, thus challenging and motivating them more, which results in more positive learning experiences and a steeper learning curve (Stipek, 2002; Hannover and Kessels, 2011). Throughout an academic career these differences might accumulate into long-lasting and stable disadvantages (e.g., lower educational attainment or labor wages) for those who started school when they were the relatively youngest in a class.

### Generalizability of relative age effects across…

The previous section dealt with relative age effects that refer to general student populations. An important issue when studying relative age effects is to examine their external validity by addressing the question of how far findings can be generalized. In doing so, it is possible to draw a much more differentiated picture and gain a deeper understanding of the factors that influence the relationship between students' relative age and their achievement. In the present study, we investigate the generalizability of relative age effects by focusing on the extent to which relative age effects hold over different student groups, time, and classes.

#### Students

Given the importance of relative age effects for educational research and policy, it is surprising that only a few studies have investigated the generalizability of relative age effects across different subgroups. Furthermore, these previous studies merely focused on differences between females and males (Black et al., 2011; Kawaguchi, 2011). However, to the best of our knowledge there are no studies on differences in relative age effects for students with and without immigrant backgrounds. Yet, this subgroup is particularly interesting and relevant since achievement and education related differences between students with and without immigrant backgrounds are well-documented internationally (i.e., OECD, 2006). Specifically, substantial achievement gaps were found throughout formal schooling: in primary (e.g., Schnepf, 2004; Kristen, 2006; Reardon and Galindo, 2009; Bos et al., 2012a,b) as well as in secondary education (e.g., OECD, 2006, 2010a,b; Prenzel et al., 2015) and at the end of formal schooling (e.g., Kristen and Granato, 2007; Diefenbach, 2009). Possible explanations for these differences might be that children from immigrant families attend preschool facilities (such as kindergarten or formal and informal day care) less often and start institutional daycare at an older age compared to non-immigrant children (Schober and Spieß, 2012; Schober and Stahl, 2014). Furthermore, immigrant children are more likely to attend lower-quality facilities (Stahl, 2015) and, even among those not attending a preschool facility, more likely to experience lower home learning quality (OECD, 2011). The same principles apply to formal schooling facilities: Students with immigrant backgrounds are more likely to attend schools with fewer resources and a high proportion of immigrant students, often in tandem with a high percentage of students from families of lower socioeconomic status. They therefore experience disadvantages with regard to learning opportunities (e.g., OECD, 2006, 2007, 2010b,c, 2015). To conclude, relative age effects may be compensated in high quality learning environments. As students with immigrant backgrounds are more likely to experience lower quality learning environments in preschool and at school, relative age effects among students with immigrant backgrounds may be more pronounced compared to students with nonimmigrant backgrounds. This prediction, however, has so far not been empirically examined.

#### Time

The question of the generalizability of relative age effects across time can be addressed (a) by longitudinally following the same cohort and studying possible changes in relative age effects as the cohort progresses through different grades, or (b) by studying possible differences among different cohorts who experience differing environmental conditions. So far, most relative age research addressing the aspect of time has taken the former approach (see Section Relative Age Effects; e.g., Crone and Whitehurst, 1999; Lincove and Painter, 2006; Gold et al., 2012). Research on the generalizability across time focusing on differences between cohorts, however, is sparse. In a study by Elder and Lubotsky (2009), a postponement of the cut-off date for enrollment by 3 months (increasing cohorts' average age) simultaneously increased academic achievement and the likelihood of repeating a grade. In this article we analyze relative age effects across different cohorts because students in each cohort were affected by different school enrollment laws. Thus, comparing relative age effects across student cohorts provides important information for policy makers and educational administrators on how school enrollment regulations may influence these effects.

#### Classes

Investigating the generalizability of relationships across schools and classes has become a popular and well-accepted empirical approach in current education research (e.g., Hill and Rowe, 1996; Cools et al., 2009; Maag Merki et al., 2015). In addition, a great deal of research has shown the effects of academic learning environments, such as the school or class a student attends, on important educational outcomes (e.g., for achievement: OECD, 2010b, 2015). Especially the class has been found to be a particularly important factor in research on students' outcomes (e.g., Martínez, 2012) because it is at this level that the differences in teaching quality, the implementation of school curricula, and thus, the handling of heterogeneous student characteristics (e.g., age) is most evident. Thus, it seems plausible that relative age effects may vary between classes, with some classes showing stronger and some classes showing weaker relative age effects.

## Research objectives and hypotheses

Relative age effects have often been investigated for the general student population in various grade levels. The generalizability of relative age effects, however, has not been fully explored. The aim of the current study is therefore to investigate achievement-related relative age effects and their generalizability across student subgroups, time, and classes. To this end, we pursue the following four research objectives. (1) We examine whether relative age effects in the general student population (as found in several previous studies) can be replicated with our data. Following the previously summarized literature, we expect that substantial relative age effects in favor of relatively older students will be found in the early grades of elementary school. Further, we expect that relative age effects will get smaller as children progress through elementary and secondary school and as relatively younger students get the chance to catch up with their relatively older peers (see Stipek, 2002). Moreover, we address three key questions on the generalizability of relative age effects. (2) In examining the generalizability across different subgroups of students, we compare general relative age effects and specific relative age effects for students with and without immigrant backgrounds. Here, we expect to find substantial differences in relative age effects between students with and without immigrant backgrounds, with relatively young immigrant students being particularly disadvantaged. In addition to the fact that they, like all children their age, are less mature and have had less time to attend preschool facilities, this group of students is further characterized by their differences with regard to education and achievement (see Section Students). (3) In order to answer the question of the generalizability of effects across time, we survey differences in relative age effects across three different student cohorts who entered school before and after state-wide reforms on school enrollment age were implemented. In doing so, we can explore whether relative age effects change (a) when the age range of a school entrance cohort is 18 in comparison to 12 months and (b) when the average age of a cohort is higher or lower. (4) Finally, given that teachers differ in their teaching quality, the implementation of school curricula, and the handling of heterogeneity amongst their students, we explore whether relative age effects are applicable to all school classes or whether students in some classes show different relative age effects than students in other classes.

## Materials and methods

### Sample

**Figure 1:**
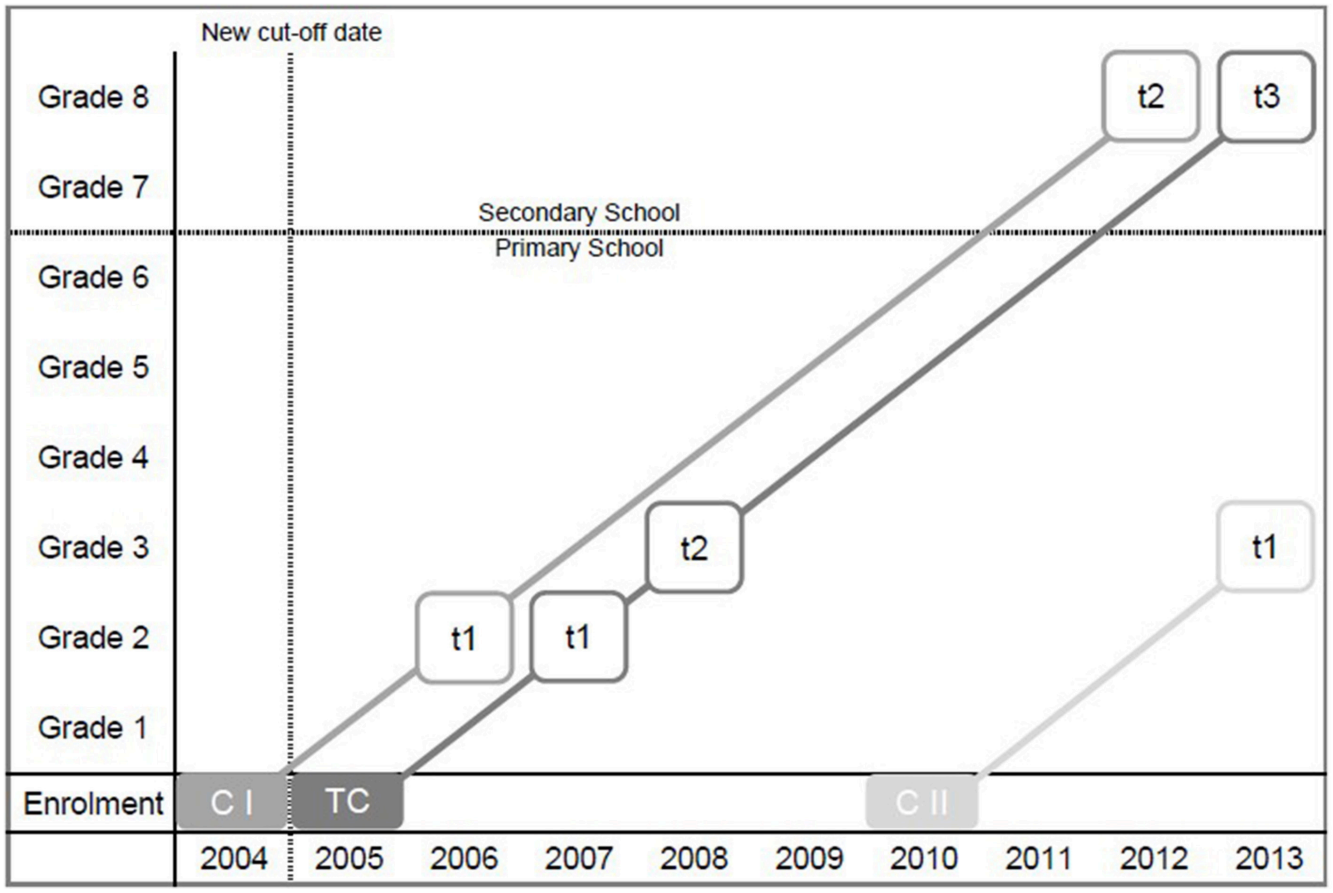
**Multi-cohort sequence design of the present study (CI, Comparison Cohort I; TC, Target Cohort; CII, Comparison Cohort II)**. *t* marks available archive data for a certain student cohort.

In our analyses, we first capitalize on representative large-scale data sets from one cohort of students who were obliged to enter public elementary school on August 1, 2005 according to the Berlin school law (see “TC” in Figure 1). In the present analyses, we have deliberately excluded any students from our analyses who entered school early or late because with the available archive data we could not control for possible selection bias among these students (e.g., influences as a result of unobserved heterogeneity due to voluntarily early enrollment or retention upon parental request). We paid special attention to this cohort, which we call the Target Cohort, because this cohort was the first to be affected by a revision of the Berlin law of school enrollment. Here, the Berlin Senate preponed the cut-off date, the date until which a child must reached 6 years of age in order to be eligible for school entry, from June, 30 to December, 31. This cohort is exceptional because it comprises three clearly distinguishable student age groups. (1) Children who were enrolled only according to the reformed (new) school entry law. These children were born between July and December 1999, and would have otherwise been enrolled a year later. We refer to this age group as young: children in this group were between 68 and 73 months old by the time they started school. (2) Children who would have been enrolled under the old and the new law of school enrollment. These children were born between January and June 1999. We refer to this age group as intermediate: children in this age group were between 74 and 79 months old at the time they started school. (3) Children who would have been enrolled according to the expiring (old) school entry law. These children were born between July and December 1998. We refer to this age group as old: students in this group were between 80 and 85 months of age at school entrance.

Taken together, the Target Cohort includes students with an average age of 6.4 years and an age range of 18 months. (Under the old law, a cohort to be enrolled at school had a mean age of 6.7 years and an age range of 12 months). Importantly, in studying the Target Cohort we have a unique opportunity to compare those students who would not have entered school yet if the reform had not been implemented with those students who would have entered school even if the reform had not been implemented, both being taught in the same learning environment. For the Target Cohort, we were able to access archive data from Grade 2 (in 2007), Grade 3 (in 2008), and Grade 8 (in 2013).

In order to investigate the generalizability of effects across time for different student cohorts, we examined two further cohorts (see “CI” and “CII” in Figure 1). We call them comparison cohorts. Comparison Cohort I includes students enrolled one year before the cut-off date was changed and thus comprises students with an average age of 6.7 years, with students being 74–85 months old when entering school (i.e., with an age range of 12 months between the youngest and the oldest children). Hence, students in Comparison Cohort I belong either to the intermediate or the old age group (as defined for the Target Cohort). For Comparison Cohort I, archive data from Grades 2 (in 2006) and 8 (in 2012) were available. Comparisons between the Target Cohort and Comparison Cohort I give insights as to whether the reform in the age of school enrollment influenced the size of relative age effects.

Comparison Cohort II, in turn, consists of students enrolled in 2011, 6 years after the new law of school enrollment became effective. Accordingly, students in this cohort were between 68 and 79 months old when they entered school, with an average age of 6.1 years and an age range of 12 months between the youngest and the oldest children. Hence, students in Comparison Cohort II belong either to the young or intermediate age group (as defined for the Target Cohort). For Comparison Cohort II archive data from Grade 3 (in 2013) was available. Comparing Cohort II with the Target Cohort allows us to test the long-term stability of relative age effects in Grade 3. For all three cohorts, we draw on archive data, which were collected in order to allow cross-sectional analyses of each cohort. Thus, we could not track individual students across time.

To determine a student's relative age, we were able to access data on age for all students of the Target Cohort in Grade 2 and Grade 3, and for students in Comparison Cohort I in Grade 2. However, information on age was not included in the archive data for some cohorts at some grade levels. We therefore drew random subsamples of students to collect these data for students in the Target Cohort and Comparison Cohort I at Grade 8, and for students in Comparison Cohort II in Grade 3 (see Table 1).

**Table 1:** **Sample description by student cohort**.

	**Comparison cohort I**				**Target cohort**				**Comparison cohort II**			
	**N**		**Male**	**MB**	**N**		**Male**	**MB**	**N**		**Male**	**MB**
	**Students**	**Classes**			**Students**	**Classes**			**Students**	**Classes**		
Grade 2	18,767	786	51.4%	32.1%	28,662	1553	51.0%	33.0%	–	–	–	–
Grade 3	–	–	–	–	25,500	1433	50.8%	27.3%	1570	112	50.3%	26.6%
Grade 8	4050	281	51.1%	32.0%	2397	122	49.8%	28.4%	–	–	–	–

*% MigB = percentage of students with immigrant backgrounds*.

### Instruments

Data for all three student cohorts stem from state-wide proficiency assessment programs where student participation was compulsory. In all programs, students worked on tests assessing proficiency in mathematics and reading comprehension. The mathematics and reading tests were based on extensive pilot studies and professional development by practitioners and psychometricians. Reliability estimates for students' proficiency scores were acceptable to good: Cronbach's Alpha for Grade 2 (Cronbach, 1951) ranged from α = 0.80 to α = 0.82. Because the tests in Grades 3 and 8 were scaled using item response theory (IRT; Wu et al., 1998), we use IRT-based estimates of reliability (Rost, 1996). These estimates ranged between 0.60 and 0.89. Note that the Berlin Ministry of Education also changed the grade levels when students take part in the assessment programs. This implies that students belonging to the Target Cohort participated in Grades 2, 3, and 8; students in Comparison Cohort I participated in Grades 2 and 8; students in Comparison Cohort II participated in Grade 3.

To assess students' immigrant backgrounds we referred to student's language background of origin or the family's lingua franca. Unfortunately, as we used archive data it was not possible to apply the same criterion for all cohorts in all grade levels. However, in data sets where both attributes were recorded at the same time the correlation coefficient exceeded *r* = 0.80, pointing to a large overlap between these attributes. Proportions of students with immigrant backgrounds ranged from 26.6% in Grade 3 of the Target Cohort up to 32.5% in Grade 2 of Comparison Cohort I and the Target Cohort.

### Analytic strategy

To study relative age effects, we used age groups (as defined above) as independent variables. The intermediate group served as the reference group because children in this group would have been enrolled under both the old and the new school law. Defining these age groups in all three student cohorts makes it possible to (a) study relative age effects within each student cohort and (b) to examine how these effects vary across cohorts that (b) entered school before (Comparison Cohort I) or (c) after the new law became effective (Comparison Cohort II). We defined groups with an age range of 6 months to balance substantive, theoretical and methodological requirements: (a) the data that we used for the present analyses included students who were enrolled only according to the old (old) or the new (young) law of enrollment and students who would have enrolled according to either law (intermediate). Thus, the definition of three age groups corresponds perfectly to the grouping of students as implied by the effect of the new enrollment law. (b) Pooling students in larger age groups is an accepted procedure in the research on relative age effects (e.g., Stipek and Byler, 2001; Cobley et al., 2009): A grouping procedure allows for the study of the potential non-linearity of relative age effects, whereas correlation-based estimates, for example, assume a linear relation between age and achievement across the whole age range. (c) We also estimated relative age effects in subsamples of students with and without immigrant backgrounds. Thus, age groups with an age range of 6 months comprise a sufficient number of students to estimate relative age effects with high precision (i.e., with small standard errors).

For the analyses contained in this paper, achievement scores were z-standardized (with *M* = 0, *SD* = 1) for mathematics and reading and for each cohort in each grade level, respectively. Doing so allowed us to compare relative age effects across cohorts, grade levels, and domains. To account for possible sampling error within the subsamples of schools for which additional information on age was collected, we (a) applied formal stratified random sampling (Shadish et al., 2001, p. 342) including achievement, district, and school type as strata and (b) weighted the collected data including population information on district, gender, migration background, and school type using the survey package (Version 3.30-3; Lumley, 2004) in R Core Team (2015). We chose class (and not school) as the clustering variable because relative age effects for an individual may depend on the instructional quality he or she experiences in his or her class (e.g., individual learning support).

To study relative age effects, we ran random intercept models for each time point and outcome variable to estimate standardized mean differences (SMDs) between age groups. We chose SMDs because they are conceptually very similar to standardized effect sizes such as Cohen's d (1992). Yet, in contrast to standardized effect sizes like Cohen's d SMDs can be directly estimated within a multilevel framework that we used in the present study. Importantly, the advantages of standardized effects sizes also apply to SMDs. More specifically, standardized effects sizes can be used to compare relative age effects across samples, study designs, and statistical methods (even when different measures were used); they can also be used to evaluate the practical relevance of effects, and they provide key information for power analyses and meta-analyses (see Wilkinson and Task Force on Statistical Inference, 1999). We used the multilevel framework as implemented in the lme4-Package (Version 1.1-8; Bates et al., 2015) in R Core Team (2015) to estimate SMDs and their standard errors. This procedure accounts for the clustering in the data (students within classes), which is necessary to compute correct standard errors. In order to estimate the precision of the results (Wilkinson and Task Force on Statistical Inference, 1999; Cumming and Finch, 2001), 95% confidence intervals (CI) were computed using the confint-function in lme4. Accordingly, we specified class as the cluster variable for each class j, achievement in mathematics or reading as the dependent variable for each student i (γ_*ij*_), and membership in the young (*young*_*ij*_) or old (*old*_*ij*_) age group (dummy-coded with the intermediate group as reference group) as independent variables (1); γ_00_ defines the intercept of the regression line and *r*_*ij*_ and *u*_0*j*_ represent random error terms on Levels 1 and 2, respectively. Doing so yielded Model Set 1a:

(1a) $$\gamma_{ij}=\;\gamma_{00}+\;\gamma_{10}old_{ij}\;+\;\gamma_{0}young_{ij}+\;u_{0j}+\;r_{ij}$$

To compute the SMD between age groups young and old, and intermediate (γ_0_*intermediate*_*ij*_) and old, a corresponding Model Set 1b was specified (with the old group as reference group):

(1b) $$\gamma_{ij}=\;\gamma_{00}+\;\gamma_{10}young_{ij}\;+\;\gamma_{0}intermediate_{ij}+\;u_{0j}+\;r_{ij}$$

We applied Model Set 1a and 1b to various samples in order to investigate our research objectives. For Research Objective 1, we drew on the overall sample of the Target Cohort. For Research Objective 2 we used the information on students' language spoken at home to subdivide the overall sample of the Target Cohort into a subsample of students with immigrant backgrounds and a subsample without immigrant backgrounds. We applied Models 1 and 2 to both subsamples separately. For Research Objective 3, we drew on data from Comparison Cohorts I and II, respectively. In contrast to the Target Cohort, however, for Comparison Cohorts I and II we did not introduce a dummy variable for the young group and old group, respectively, because these groups did not exist in these cohorts.

To address Research Objective 4 on the generalizability of relative age effects across classes, we first ran random-intercept models on the basis of the Target Cohort as specified in Equation (1) (see above):

(2.1) $$\gamma_{ij}=\;\gamma_{00}+\;\gamma_{10}old_{ij}\;+\;\gamma_{0}young_{ij}+\;u_{0j}+\;r_{ij}$$

Subsequently, we allowed the age effect for the old group to vary across classes in Model Set 2 and estimated a variance component for the regression coefficient *u*_1*j*_:

(2.2) $$\gamma_{ij}=\;\gamma_{00}+\;\gamma_{10}old_{ij}\;+\;\gamma_{0}young_{ij}+\;u_{0j}+u_{1j}old_{ij}+\;r_{ij}$$

In Model Set 3, we additionally allowed the age effect for the young group to vary across classes and estimated a variance component for the regression coefficient *u*_2*j*_:

(2.3) $$\begin{array}{l} \gamma_{ij}=\;\gamma_{00}+\;\gamma_{10}old_{ij}\;+\;\gamma_{0}young_{ij}+\;u_{0j}+u_{1j}old_{ij}\\ \;+\;u_{2j}young_{ij}\;+\;r_{ij} \end{array}$$

By executing deviance tests that inspected whether a model fits the data better than its preceding model, we examined whether the relative age effects applied for all classes in a similar way.

## Results

### Descriptive statistics

Before proceeding to the results for our research questions, we are going to take a short look at the distribution of the three age groups. Although we aimed to keep selection bias at a minimum by referring to only those students who were obliged to enter school in a certain year (see Section Sample), we found that the group of relatively older students includes a smaller number of students on average in comparison to the other age groups. This is due to the fact that some parents voluntarily enrolled their child a year earlier. In the year before the Target Cohort entered school, ~5.5% of the children were enrolled early (Autorengruppe Bildungsberichterstattung, 2012), meaning that the group of relatively older students in the Target Cohort is to some extent selected (e.g., because these students might have shown exceptionally good precursory verbal or mathematical skills or appeared to be quite mature for their age). For the year before Comparison Cohort I started school, ~3% of children were enrolled early. In Comparison Cohort II, around 1% of students were enrolled early (Autorengruppe Bildungsberichterstattung, 2012).

For students with immigrant backgrounds (column MigB in Table 2), the proportions are distributed similarly, with the proportion of the old within the students with immigrant backgrounds being a little bit bigger than in the group of all students. When focusing on changes in the proportions over time, we found that it is the group of the young and especially the young with immigrant backgrounds whose percentages decrease the most over the grades (from 35.5% in Grade 2 to 30.2% in Grade 8 for the Target Cohort, Table 2).

**Table 2:** **Proportions of the three age groups with respect to the overall student sample and within subgroups of students with and without immigrant backgrounds by student cohort**.

**Age groups**	**Comparison cohort I**			**Target cohort**			**Comparison cohort II**		
	**Total**	**No MigB**	**MigB**	**Total**	**No MigB**	**MigB**	**Total**	**No MigB**	**MigB**
**GRADE 2**									
Young	–	–	–	35.5%	35.6%	35.4%	–	–	–
				[34.9, 36.1]	[34.8, 36.3]	[34.4, 36.5]			
Intermediate	52.6%	52.9%	52.1%	36.5%	36.8%	35.9%	–	–	–
	[51.8, 53.5]	[51.9, 53.9]	[50.8, 53.3]	[35.9, 37.1]	[36, 37.5]	[34.9, 36.9]			
Old	47.4%	47.1%	47.9%	28.1%	27.7%	28.7%	–	–	–
	[46.5, 48.2]	[46.1, 48.1]	[46.7, 49.2]	[27.4, 28.7]	[27, 28.4]	[27.7, 29.6]			
Total N	18,767	12,745	6022	28,662	19,177	9458			
**GRADE 3**									
Young	–	–	–	33.6%	34%	32.9%	47.4%	47.5%	47.1%
				[33, 34.3]	[33.2, 34.7]	[31.6, 34.1]	[44.9, 53.2]	[44.6, 54.2]	[42.3, 52.9]
Intermediate	–	–	–	37.6%	37.5%	37.7%	52.6%	52.5%	52.9%
				[36.9, 38.2]	[36.7, 38.2]	[36.6, 38.9]	[50.1, 50.2]	[49.6, 49.9]	[48, 52.9]
Old	–	–	–	28.8%	28.6%	29.5%	55.1%	55.4%	57.7%
				[28.2, 29.5]	[27.8, 29.3]	[28.3, 30.7]	–	–	–
Total N				25,500	18,550	6950	1570	1152	418
**GRADE 8**									
Young	–	–	–	31.4%	31.9%	30.3%	–	–	–
				[29.5, 33.4]	[29.6, 34.2]	[26.5, 33.9]			
Intermediate	51.5%	52.1%	50%	39.6%	39.3%	40.4%	–	–	–
	[49.8, 53.2]	[50.1, 54.2]	[47.1, 52.9]	[37.6, 41.6]	[36.8, 41.7]	[36.7, 44.1]			
Old	48.5%	47.9%	50%	29%	28.7%	29.2%	–	–	–
	[46.8, 50.2]	[45.8, 49.9]	[47.1, 52.9]	[26.9, 31]	[26.2, 31.2]	[25.7, 32.6]			
Total N	4050	2755	1295	2397	1717	680			

*Total, general sample of students; no MigB, students without immigrant backgrounds; MigB, students with immigrant backgrounds; 95% confidence intervals are displayed in square brackets*.

### Relative age effects

Table 3 (“*Target Cohort*”) displays the relative age effects for the Target Cohort. The results for reading in Grade 2 show effects to the advantage of the relatively older students. The effect is largest between young and old students (SMD_Reading_ = −0.19) and smallest between intermediate and old (SMD_Reading_ = −0.04). For mathematics, the effects are identical, albeit more distinctive (young vs. old: SMD_Mathematics_ = −0.23; intermediate vs. old: SMD_Mathematics_ = −0.07).

**Table 3:** **Relative age effects (standardized mean differences) as observed for the overall student samples by student cohort**.

**Age groups**	**Comparison cohort I**		**Target cohort**			**Comparison cohort II**
	**Grade 2**	**Grade 8**	**Grade 2**	**Grade 3**	**Grade 8**	**Grade 3**
**READING ACHIEVEMENT**						
Young vs. intermediate			−0.14	−0.09	0.06	−0.14
			[−0.17, −0.12]	[−0.12, −0.07]	[−0.01, 0.13]	[−0.23, −0.05]
Intermediate vs. old	−0.02	0.06	−0.04	0.01	0.04	
	[−0.05, 0.01]	[0.01, 0.11]	[−0.07, −0.02]	[−0.02, 0.04]	[−0.03, 0.11]	
Young vs. old			−0.19	−0.09	0.10	
			[−0.21, −0.16]	[−0.12, −0.07]	[0.03, 0.17]	
**MATHEMATICS ACHIEVEMENT**						
Young vs. intermediate			−0.16	−0.12	0.00	−0.15
			[−0.19, −0.14]	[−0.14, −0.09]	[−0.07, 0.07]	[−0.24, −0.05]
Intermediate vs. old	−0.04	−0.03	−0.07	−0.02	0.05	
	[−0.07, −0.02]	[−0.13, 0.07]	[−0.09, −0.04]	[−0.04, 0.01]	[−0.02, 0.12]	
Young vs. old			−0.23	−0.13	0.05	
			[−0.25, −0.2]	[−0.16, −0.11]	[−0.03, 0.12]	

*95%-confidence intervals are displayed in square brackets. Negative values indicate that the mean achievement level of the group comprising younger students (e.g., young) is lower than that mean achievement level of the group comprising older students (e.g., intermediate)*.

In Grade 3, effects in reading and mathematics between young and intermediate as well as between young and old are somewhat smaller than in Grade 2, but still in favor of relatively older students (except for the comparison of the intermediate vs. old group).

Finally, in Grade 8 relative age effects have vanished in the general sample in reading and mathematics or have even been reversed. Specifically, in reading, students in the young group now outperform students in the old group (SMD_Reading_ = 0.10).

In sum, the present results show that relative age effects on reading achievement and on mathematics achievement in favor of older students are most pronounced in Grade 2 and diminish (or even reverse in favor of younger students) with increasing grade levels.

### Generalizability of relative age effects across…

The following subsections tackle questions on the extent to which the results as observed for the overall sample in the Target Cohort can be generalized across students with and without immigrant backgrounds, time (i.e., different cohorts), and classes.

#### Students

Table 4 presents the results for the immigrant and the non-immigrant samples, respectively. The only substantial difference between students with and without immigrant backgrounds, which we detected by way of non-overlapping confidence intervals, is for young vs. old in Grade 2 reading. Besides that, the present results suggest that the results as observed for the overall sample can also be generalized to both students with immigrant backgrounds and students without immigrant backgrounds.

**Table 4:** **Relative age effects (standardized mean differences) for students with and without immigrant backgrounds**.

**Age groups**	**No migration backgrounds**			**With migration backgrounds**		
	**Grade 2**	**Grade 3**	**Grade 8**	**Grade 2**	**Grade 3**	**Grade 8**
**READING ACHIEVEMENT**						
Young vs. intermediate	−0.14	−0.09	0.04	−0.16	−0.11	0.09
	[−0.17, −0.11]	[−0.12, −0.06]	[−0.04, 0.12]	[−0.2, −0.11]	[−0.16, −0.06]	[−0.04, 0.21]
Intermediate vs. old	−0.02	0.01	0.03	−0.09	0.00	0.08
	[−0.05, 0.01]	[−0.02, 0.04]	[−0.06, 0.11]	[−0.14, −0.04]	[−0.05, 0.05]	[−0.05, 0.21]
Young vs. old	−0.16	−0.08	0.06	−0.25	−0.11	0.16
	[−0.19, −0.13]	[−0.11, −0.05]	[−0.02, 0.15]	[−0.3, −0.2]	[−0.16, −0.06]	[0.02, 0.3]
**MATHEMATICS ACHIEVEMENT**						
Young vs. intermediate	−0.18	−0.12	−0.02	−0.14	−0.14	0.03
	[−0.2, −0.15]	[−0.14, −0.09]	[−0.1, 0.06]	[−0.17, −0.1]	[−0.19, −0.1]	[−0.09, 0.16]
Intermediate vs. old	−0.04	−0.01	0.04	−0.11	−0.03	0.08
	[−0.08, −0.01]	[−0.04, 0.02]	[−0.04, 0.12]	[−0.15, −0.07]	[−0.08, 0.02]	[−0.05, 0.21]
Young vs. old	−0.22	−0.13	0.02	−0.24	−0.17	0.12
	[−0.25, −0.19]	[−0.16, −0.09]	[−0.07, 0.11]	[−0.29, −0.2]	[−0.22, −0.12]	[−0.02, 0.26]

*Results refer to the Target Cohort. 95% confidence intervals are displayed in square brackets. Negative values indicate that the mean achievement level of the group comprising younger students (e.g., young) is lower than that mean achievement level of the group comprising older students (e.g., intermediate)*.

#### Time

The results from the study on the generalizability of relative age effects across different cohorts are displayed in Table 3 (“*Comparison Cohort I*”, “*Comparison Cohort II*”). For Comparison Cohort II, the results from the Target Cohort were replicated in both reading and mathematics with no substantial differences in size. For Comparison Cohort I, the results were also replicated for mathematics, but not for reading. Here, there is already a zero effect in Grade 2 (SMD_Reading_ = −0.02) and a positive effect for the relatively younger (here: intermediate) in Grade 8 (SMD_Reading_ = 0.06). However, none of the standardized mean differences in Comparison Cohorts I and II are substantially different from the general relative age effects found for the Target Cohort.

#### Classes

In our last set of analyses, we studied the generalizability of age effects across classes. The results for reading in Grade 2 (Table 5) show that a model including a random coefficient for the age group old (Model Set 2) fits the data significantly better than a model where there are only fixed effects specified (Model Set 1). However, these significant results are likely due to the large sample size, which may render even trivial differences statistically significant. Moreover, when a random coefficient is further added for the young (Model Set 3), no significant changes in model fit are found. Further, focusing on the size of effects, the close to zero variance of the random coefficients (e.g., for Grade 2: $\tau_{young}^{2}$ = 0.02 and $\tau_{old}^{2}$ = 0.01) indicates that there is no substantial variation in relative age effects across classes. These results apply for reading in all grades studied. For mathematics, the results are similar, implying no systematic variation in general relative age effects across classes.

**Table 5:** **Relative age effects (random coefficients models) across classes**.

	**Parameter estimates**						**Variance components**			**Model comparison (deviance tests)**					
	**Intercept**		**Young**		**Old**		**Intercept**	**Young**	**Old**	**Compare**	***Δχ*^2^**	**p**	**df**	**AIC**	**BIC**
	**Estimate**	**SE**	**Estimate**	**SE**	**Estimate**	**SE**	**Estimate**	**Estimate**	**Estimate**						
**READING ACHIEVEMENT**															
**GRADE 2**															
Model 1	0.01	0.02	−0.14	0.01	0.04	0.01	0.25	–	–				5	72,689	72,731
Model 2	0.01	0.02	−0.14	0.01	0.04	0.01	0.26	–	0.00	M2 vs. M1	12.75	0.002	7	72,681	72,738
Model 3	0.01	0.02	−0.14	0.01	0.05	0.01	0.26	0.02	0.01	M3 vs. M2	3.86	0.277	10	72,683	72,765
**GRADE 3**															
Model 1	0.01	0.02	−0.09	0.01	−0.01	0.01	0.34	–	–				5	63,854	63,894
Model 2	0.01	0.02	−0.09	0.01	−0.01	0.01	0.34	–	0.02	M2 vs. M1	8.78	0.012	7	63,849	63,906
Model 3	0.01	0.02	−0.09	0.01	−0.01	0.01	0.34	0.00	0.02	M3 vs. M2	0.36	0.948	10	63,855	63,936
**GRADE 8**															
Model 1	−0.14	0.07	0.11	0.03	0.02	0.03	0.53	–	–				5	6098.2	6127.7
Model 2	−0.14	0.07	0.11	0.03	0.02	0.03	0.56	–	0.04	M2 vs. M1	10.42	0.005	7	6091.8	6133.1
Model 3	−0.14	0.07	0.12	0.03	0.02	0.04	0.59	0.00	0.04	M3 vs. M2	4.42	0.219	10	6093.3	6152.4
**MATHEMATICS ACHIEVEMENT**															
**GRADE 2**															
Model 1	0.00	0.02	−0.16	0.01	0.07	0.01	0.36	–	–				5	69,244	69,285
Model 2	0.00	0.02	−0.16	0.01	0.07	0.01	0.36	–	0.00	M2 vs. M1	1.77	0.412	7	69,246	69,303
Model 3	0.00	0.02	−0.16	0.01	0.07	0.01	0.37	0.00	0.00	M3 vs. M2	0.15	0.985	10	69,252	69,334
**GRADE 3**															
Model 1	0.01	0.02	−0.12	0.01	0.02	0.01	0.34	–	–				5	64,056	64,097
Model 2	0.01	0.02	−0.12	0.01	0.02	0.01	0.34	–	0.01	M2 vs. M1	2.43	0.297	7	64,058	64,115
Model 3	0.01	0.02	−0.12	0.01	0.02	0.01	0.34	0.00	0.01	M3 vs. M2	0.89	0.827	10	64,063	64,144
**GRADE 8**															
Model 1	−0.15	0.07	0.04	0.03	−0.01	0.03	0.55	–	–				5	5850.9	5880.3
Model 2	−0.15	0.07	0.04	0.03	−0.01	0.03	0.57	–	0.00	M2 vs. M1	2.80	0.246	7	5852.1	5893.3
Model 3	−0.15	0.07	0.04	0.04	0.00	0.03	0.59	0.02	0.01	M3 vs. M2	2.11	0.550	10	5856	5914.8

*Results refer to the Target Cohort. SE, standard error; Δχ^2^, change in chi-square; p, probability of the observed deviance difference given that the deviance between models is zero in the population; df, degrees of freedom; AIC, Akaike information criterion; BIC, Bayesian information criterion*.

## Discussion

The overarching goal of the present study was to tackle key questions regarding the replicability and generalizability of relative age effects on students' achievement with respect to (a) students with and without immigration backgrounds, (b) time in terms of reforms to school enrollment regulations, and (c) across classes. To this end, we capitalized on a quasi-experimental research situation that resulted from an educational reform as well as a unique set of population and large-scale data from students in Berlin, the capital of Germany.

### Relative age effects

We examined whether previous empirical evidence on general relative age effects could be replicated with the overall sample for the Target Cohort. Based on previous research, we hypothesized that substantial relative age effects in favor of relatively older students would be found in the early grades of elementary school (Grade 2), as well as that these effects will get smaller (Grade 3), and eventually vanish (Grade 8) over the course of academic education. Our results generally confirm these predictions for reading and mathematics. We even found a significant reversed relative age effect favoring the young over the old in reading in Grade 8.

To further evaluate the relative age effects as found in the present study, we refer to the seminal paper by Hill et al. (2008, see Table 1), who meta-analyzed annual achievement gains in reading and mathematics for the K-12 age range. In Grade 2, the differences between the youngest and the oldest students of that cohort can be quantified as a fifth of the average annual gain in reading and mathematics, which means that the oldest were on average 10 weeks ahead in their achievement compared to the youngest. As the cohorts proceeded through Grade 3, the differences decreased to an advantage of a little more than 4 weeks in reading and ~7 weeks in mathematics. In Grade 8 the only substantial relative age effect pointed to a 20-week advantage among the youngest in comparison to the oldest in reading.

### Generalizability of relative age effects across…

After studying relative age effects in general we proceeded to examine their generalizability.

#### Students

We examined the generalizability of general effects across students with and without immigrant backgrounds. We hypothesized stronger relative age effects to the disadvantage of young immigrants (e.g., because of inequalities in preschool experiences). The findings provided some support for this prediction for reading in Grade 2, where the differences represent an 8-week advantage for the oldest in comparison to the youngest Germans and a 12 week advantage for the oldest in comparison to the youngest students with immigrant backgrounds. However, substantial differences in relative age effects between students with and without immigrant backgrounds were not observed with the available data for either Grade 3 or Grade 8. The general trend of the relative age effects decreasing over time is similar for all subsamples. However, particularly interesting here is that in contrast to their German peers, relative age effects to the disadvantage of young students with immigrant backgrounds did not yet show a substantial decrease by Grade 3. This finding might indicate that younger immigrants need more time to catch up with their older peers than the younger Germans do.

#### Time

With regard to the question on the generalizability of relative age effects across time, we surveyed differences in relative age effects between three different school entrance cohorts (2004, 2005, and 2010). In doing so, we were able to explore whether relative age effects change (a) when the age range of a school entrance cohort is 18 months rather than 12 and (b) when the average age of a cohort is higher or lower. Specifically, in Comparison Cohort I, the age average was 6.7 years and the age range was 12 months; in the Target Cohort, the average age was 6.4 years with a range of 18 months; and in Comparison Cohort II, the average age was 6.1 years with a range of 12 months.

Importantly, relative age effects could be generalized across the three cohorts in mathematics. In reading, similar effects were found for the Target Cohort and Comparison Cohort II, a cohort that started school 6 years after the Target Cohort. This points to the sustainability of the findings as observed for the Target Cohort. In contrast to the Target Cohort, however, students in Comparison Cohort I showed no relative age effect in Grade 2 and a positive effect for the relatively young (here: intermediate) in Grade 8, as opposed to a negative effect for the young in Grade 2 and zero effect in Grade 8 for students in the Target Cohort. A potential explanation for the “missing” relative age effects in Grade 2 might be that the relatively younger in Comparison Cohort I are still older (and hence more mature) than the young in the Target Cohort and thus able to achieve at a similar level to their older peers.

In summary, relative age effects in mathematics and reading did not differ substantially between student cohorts and can thus be generalized to student cohorts with a higher average age and a larger age range. Moreover, the reversal of relative age effects (which was observed for both the Target Cohort and Comparison Cohort I in reading) is particularly interesting because it shows that the youngest children's disadvantage in reading achievement in Grade 2 might turn into an advantage in Grade 8 (but see also Limitations Section).

#### Classes

We investigated whether relative age effects are applicable to all school classes or whether students in some classes show different relative age effects than students in other classes. Here, we expected to find substantial variation in effects between classes. Our results indicate that relative age effects did not vary substantially across classes in reading and mathematics. In other words: Even though attending a certain class or learning group is known to be an important indicator in education research (e.g., Martínez, 2012), the different learning environment within each class did not seem to substantially affect the effects of a child's relative position according to her or his age on achievement in public schools in Berlin.

### Limitations

In our analyses, we examined a unique set of data that covered the whole student population or representative samples of student achievement data in reading and mathematics. However, the present data did not allow us to track individual students across time, which limited our opportunities for studying the reasons why students dropped out of the sample. It is possible that the vanishing or even the reversal of relative age effects results from younger students with a lower level of achievement being retained at lower grade levels. However, the proportion of students with each age group changed only slightly as students proceeded through their educational careers: For example, the proportion of students in the young group in the Target Cohort dropped from 35.5% in Grade 2 to 33.4% in Grade 8 (see Table 1). Thus, it seems unlikely that this explanation fully accounts for the pattern of results on relative age effects as observed in the present study. To overcome this limitation, future studies on relative age effects may benefit considerably from (a) tracking all students who enter school in a certain school year irrespective of whether they are retained at a certain grade level or not and (b) collecting data on a broad range of students' individual (e.g., early number or verbal competencies, affective-motivational variables) and family characteristics (e.g., parents' educational aspirations for their children) that are known to predict whether students linearly progress through their educational career or not. Finally, to get a fuller picture of relative age effects, it would be beneficial to include further outcome variables, particularly non-cognitive ones (e.g., social skills), because these outcomes receive a great deal of attention in the public and scientific discussion of school starting age (see Stipek, 2002).

### Concluding remarks

In sum, the results of our study confirm the pattern of findings on relative age effects as identified by the majority of previous studies: Relative age effects are relatively small at the beginning of formal schooling and largely vanish as students proceed through their educational careers. Moreover, our study has provided new evidence on the wide generalizability of these findings. Although we found a few differences, overall the present study has empirically underscored that the pattern of results as observed for the overall sample in the Target Cohort holds (a) for students with and without immigrant backgrounds; (b) for student cohorts who entered school under differing enrollment regulations, resulting in variations in the average age and age range; and (c) across school classes. Importantly, the size of these relative effects, especially in earlier grades, adds to the empirical body of knowledge on the heterogeneity that exists among students, not only but especially in elementary schools. These relative age effects are particularly relevant for teachers and policy makers as they present a challenge with regard to age-appropriate instruction and curriculum development. On the other hand, findings from secondary schools support the school's role as the “great equalizer” (e.g., Downey et al., 2004; Kerstan and Spiewak, 2008), as school minimizes the effect of children's relative age in their academic future.

## Ethic statement

Data from the standardized tests and additional subsamples were collected according to Berlin regulation on academic quality assurance and evaluation (EvalV BE). The administration of these standardized tests was approved by the Berlin Senate of Education, Youth, and Science. For the article we were able to use these existing (anonymized) data for the purpose of the study. Data for the additional subsamples were primarily collected for the purpose of the present study. The data collection was also approved by the Berlin Senate of Education, Youth, and Science.

## Author contributions

KT, EH, and MB substantially contributed to the conception of the work, interpretation of data for the work, revising the work critically for important intellectual content as well as approved the final version of the work to be published and agree to be accountable for all aspects of the work in ensuring that questions related to the accuracy or integrity of any part of the work are appropriately investigated and resolved. KT developed the general notion and wrote most of this article. She further specified the models and ran of analyzes. MB is the first supervisor of KTs dissertation and contributed on all parts of the article: discussing the general notion, the literature, the pros and cons of different methodological approaches, how to display the results and their implications.

### Conflict of interest statement

The authors declare that the research was conducted in the absence of any commercial or financial relationships that could be construed as a potential conflict of interest.
